# Periodontal Bifunctional Biomaterials: Progress and Perspectives

**DOI:** 10.3390/ma14247588

**Published:** 2021-12-10

**Authors:** Qiuxia Huang, Xin Huang, Lisha Gu

**Affiliations:** 1 Department of Operative Dentistry and Endodontics, Guanghua School of Stomatology, Hospital of Stomatology, Sun Yat-sen University, Guangzhou 510055, China; huangqx28@mail2.sysu.edu.cn; 2 Guangdong Provincial Key Laboratory of Stomatology, Sun Yat-Sen University, Guangzhou 510055, China; 3 Department of Periodontology, Guanghua School of Stomatology, Hospital of Stomatology, Sun Yat-sen University, Guangzhou 510055, China

**Keywords:** periodontitis, antibacterial, bone regeneration, biomaterials

## Abstract

Periodontitis is a chronic infectious disease that destroys periodontal supportive tissues and eventually causes tooth loss. It is attributed to microbial and immune factors. The goal of periodontal therapy is to achieve complete alveolar bone regeneration while keeping inflammation well-controlled. To reach this goal, many single or composite biomaterials that produce antibacterial and osteogenic effects on periodontal tissues have been developed, which are called bifunctional biomaterials. In this review, we summarize recent progress in periodontal bifunctional biomaterials including bioactive agents, guided tissue regeneration/guided bone regeneration (GTR/GBR) membranes, tissue engineering scaffolds and drug delivery systems and provide novel perspectives. In conclusion, composite biomaterials have been greatly developed and they should be chosen with care due to the risk of selection bias and the lack of evaluation of the validity of the included studies.

## 1. Introduction

Periodontitis is a common chronic infectious disease, and severe periodontitis affects 10.8% of the population in the world [1]. It is the primary cause of tooth loss in adults [2]. It causes irreversible damage to periodontal soft and hard tissues, including alveolar bone. Furthermore, it is closely related to many systemic diseases, including cardiovascular disease, diabetes, etc. [3]. The etiology of periodontitis is complex, involving host response, genetic, environmental and microbial factors [4]. A recent study suggested that vitamin D levels might be related to periodontitis [5] and a pilot study shed light on further exploring the link between metabolic syndrome and periodontitis [6]. Overall, the generally accepted pathogenesis is an imbalance of oral symbiotic microbial groups, along with host immune defense [4]. Moreover, the accumulation of bacteria related to periodontitis hinders tissue healing and regeneration processes [7,8].

Many efforts have been made to improve periodontal treatment outcome. Typical treatment of periodontitis focuses on pathological process and reaches a satisfactory outcome [9,10,11]. However, effectively regenerating periodontal tissues remains a challenge [12]. The purpose of periodontal therapy is to realize periodontal tissue regeneration after controlling inflammation via infection clearance [13]. To reach this goal, bioactive materials with antibacterial and osteogenic properties are in constant evolution. They are classified as bioactive agents, guided tissue regeneration/guided bone regeneration (GTR/GBR) membranes, tissue engineering scaffolds and drug delivery systems according to different patterns of action. Bioactive agents and drug delivery systems may be applied under the gingiva. GTR/GBR membranes are barriers covering periodontal defect and protecting the osteogenesis space from epithelial invasion. Tissue engineering scaffolds are applied to fill the periodontal defect. The purpose of this review is to summarize antibacterial and osteogenic performances, and advantages and limitations of different bifunctional biomaterials, and to provide novel perspectives for further study.

## 2. Biomaterials

Periodontal biomaterials fight against pathogenic bacteria and promote alveolar bone reconstruction in lesions or bone defects. These biomaterials have different forms, such as agents, membranes, particles, pastes and gels. All these materials are used alone or in combination, with different performances, advantages and limitations in periodontal treatments. The summary of periodontal biomaterials is shown in Table 1.

### 2.1. Bioactive Agents

Periodontitis is a complex chronic disease, and comprehensive agents with different functions are of great demand for treatment [14]. Bioactive agents are either biological or synthetic, with both antibacterial and osteogenic activity. As potential therapeutic agents, they are prospective to improve the outcome of periodontal treatment. 

In vitro and in vivo experiments, and even clinical trials have proven bioactive agents’ effects. In vitro experiments showed that they presented antibacterial activity against periodontal pathogens such as *Porphyromonas gingivalis* (*P. gingivalis*) and *Streptococcus mutans* (*S. mutans*). In addition, they promoted proliferation or osteogenic differentiation of cells responsible for periodontal regeneration, including human periodontal ligament fibroblasts (hPDLFCs), human periodontal ligament cells (hPDLCs) and bone marrow mesenchymal stem cells (BMSCs) [8,11,14,15]. In a mouse periodontitis model, psoralen and angelicin were able to reduce bone loss [14]. Particularly, 0.75% boric acid gel (BA) was placed under the gingiva as an adjunct to mechanical therapy in a clinical trial [15]. The results demonstrated that BA could generate some benefits, regarding clinical and radiographic indices. Furthermore, some agents could regulate inflammation and immune response, showing antioxidant activity [11,14,15]. 

Although bioactive agents have some advantages including safety, bifunction and convenient access, there are some limitations. For example, the efficacy of specific ingredients in some agents remains a mystery. To promote clinical application, further research is required to investigate the mechanisms and action modes in periodontal healing and regeneration processes. Further research is also necessary to be carried out in vivo.

### 2.2. GTR/GBR Membranes

The GTR/GBR membrane functions as a cell barrier to prevent the invasion of epithelium and connective tissues, facilitating the regeneration of periodontal ligament, cementum and alveolar bone [16]. At present, commercially available GTR/GBR membranes possess poor biocompatibility, poor stability and mechanical properties, fast degradation rate, and limited antibacterial and regeneration ability [16,17,18,19]. In the process of bone healing, infection caused by the colonization of pathogens at the defect site is one of the main causes of GBR failure [20]. To overcome these drawbacks, composite GTR/GBR membranes with different antibacterial and osteogenesis components have been designed, and present different performances [18,21,22]. They have great potential for periodontal treatment and may become promising alternative materials for GTR/GBR.

The antibacterial components of the GTR/GBR membranes mainly include antibiotics [23,24,25,26], nanoparticles of metals and their compounds [9,17,18,21,22,27], antimicrobial peptides (AMP) [28], and others. In clinic, antibiotics are commonly applied to resist the infection caused by oral pathogens and maintain a good healing environment. When encapsulated in inorganic nano carriers, their toxicity can be reduced [26]. In the microenvironment, a slow release of antibiotics can be maintained, as well as local effective concentration [20]. At present, minocycline [20], azithromycin [29], amoxicillin [23,30], doxycycline (DOX) [24], ornidazole [25] and metronidazole [26] are the main antibiotics used in the composite membranes. However, bacterial resistance of antibiotics has urged scientists to consider new antibacterial components [29]. Due to excellent antibacterial performance and little resistance, nanoparticles of metals and their compounds such as silver nanoparticles (AgNPs) and AMP have caught people’s attention [9,17,18,21,22,27]. Both of them rarely induce bacterial resistance due to multiple mechanisms. For example, they release metal ions, destroy the cell wall and membrane, penetrate the membrane and then disrupt intracellular activity including the synthesis of DNA, RNA and protein, etc., and stimulate the generation of activated oxygen [31,32,33]. Other antibacterial ingredients include polymers such as chitosan (CS) [28,34,35], traditional Chinese medicines such as curcumin [36] and inorganic materials such as bioactive glass [35].

The osteogenic components of GTR/GBR membranes mainly include conventional drugs, metal ions, inorganic materials and proteins, etc. They directly or indirectly regenerate bone in the following ways: (1) promoting migration, adhesion, proliferation and osteogenic differentiation of bone progenitor cells, (2) improving the expression level of osteogenic related genes and proteins, (3) promoting mineral deposition. Additionally, minocycline could reduce bone resorption [20]. Apart from bioactive ingredients, surface topography of the membranes which is beneficial to adhesion and spread of osteogenic-related cells could also enhance new bone formation. Lian et al. fabricated a composite membrane which was based on poly(lactic-co-glycolic acid)/gelatin (PG) fiber matrix and contained copper-loaded mesoporous silica nanoparticles (Cu@MSNs). It was suggested that the osteogenic ability of the membrane was better than that of frequently used membranes in clinics [37].

Apart from osteogenic properties, some composite membranes also had other abilities. For example, they showed anti-inflammatory performance [17,23,27,29,38] and had the ability to promote angiogenesis [20,37]. It is necessary to control inflammation since periodontal tissues are difficult to repair in an inflammatory microenvironment [39]. In addition, blood vessels can bring oxygen and blood to the lesion, which are important for bone tissue reconstruction [20]. Therefore, these multifunctional composite membranes may create a more favorable environment for periodontal tissue healing. To achieve both hard and soft tissue regeneration, asymmetric GBR films with dense and loose layers were synthesized [20,24,36,37]. The dense layer prevented fibrous connective tissue from invading the defect space, promoting fibroblast adhesion so as to enhance the formation of soft tissue above the new bone [36]. While the loose layer, which directly contacted with the bone defect space, was conducive to osteoblast adhesion, penetration and blood clot stability, thus guiding bone regeneration [37]. Moreover, a polylactide copolymer membrane showed comparable performance in mucosal healing and bone formation compared with a validated membrane for keratinized oral mucosa healing [40]. This may be attributed to the multiple properties of lactic acid, including tissue regeneration and antiseptic aspects.

Accurate matching and personalized customization are highlights of GTR/GBR membranes. Conventional GTR/GBR membranes need to be tailored according to various defects before implantation, resulting in inaccurate matching. In addition, different individuals have different antibacterial and osteogenic stages in the process of periodontal tissue healing. Considering these facts, Xu et al. designed an injectable hydrogel composite membrane [17]. It was able to automatically match the bone defect area by liquid and solid phase transformation. Therefore, it greatly improved operability and matching accuracy of the bone defect. Under the irradiation of blue light, the compound achieved early debridement of bacteria and produced Cu^2+^ with osteogenic properties. Under near-infrared irradiation, the composite produced a photothermal effect, and enhanced bone regeneration along with Cu^2+^. Through dual-light noninvasive regulation, the complex could transform antibacterial and osteogenic effects for patients at different healing stages. In conclusion, it could be a promising customized GTR strategy according to different defects and patients.

Currently, stability of the wound is considered to be crucial to periodontal regeneration as opposed to physical obstruction. A noninferiority study supported this view [41]. Platelet-rich fibrin (L-PRF) is a biological mediator with antibacterial effect against *P. gingivalis* but without a stable barrier effect [42]. Combined with inorganic bovine bone graft (IBB), L-PRF shows noninferior efficacy regarding clinical attachment level gain compared with collagen membrane in unfavorable infrabony defects. The effect of L-PRF contributed to maintenance of bone graft and fibrin, along with released polypeptide growth factors that stimulated regeneration. However, the contribution of antimicrobial properties of L-PRF was not mentioned here.

Compared with the traditional barrier membrane, novel GTR/GBR membranes have many improved properties and limitations. These properties include good biocompatibility [20], better mechanical property [37], controllable biodegradability, drug encapsulation ability, satisfactory antibacterial and osteogenesis ability [20], manageability as well as accurate matching with the defects [17]. The limitations are as follows. (1) Long degradation time. It was suggested that membranes generally need to maintain the barrier function for four–six weeks to ensure the repair of periodontal tissues in clinic [43,44]. However, according to available data, degradation time of newly developed membranes varies from about two months to more than six months [9,17,20,21,25,26,36,38]. In these circumstances, the membrane occupies the repair space of periodontal tissue and may affect complete restoration. (2) Long-term degradation and toxicity of elements. Studies have shown that the biocompatibility of the composite membranes was affected by high concentration of some elements, such as Cu^2+^ [37], TiO_2_ [17], Co^2+^ [38], nMgO [18], ornidazole [25] and metronidazole [26]. Therefore, it is necessary to further verify their long-term metabolic effect and toxicity.

### 2.3. Tissue Engineering Scaffolds

Tissue engineering scaffolds play an important role in maintaining space, storing growth factors and supporting cell attachment and proliferation after being grafted to the bone defect [39]. Three key factors of tissue engineering are the scaffold, cells and signals [16]. Ideal bone graft materials should have osteoconductivity, osteoinduction and osteogenesis properties. They allow migration of osteogenic cells, differentiation of stem cells and new bone formation, respectively. However, existing bone graft materials are osteoconductive, but lack sufficient osteoinductivity, stable bone regeneration and antimicrobial properties. Moreover, xenogenic and allogeneic bone grafts even create a risk of infection [45]. To make some improvements, tissue engineering scaffolds are developed in combination with growth factors or stem cells, as well as antibacterial components. These scaffolds have improved osteoinduction, osteogenesis and antibacterial activity.

Antibiosis is a new development of tissue engineering scaffolds. Apart from antibiotics, CS and bioglasses were incorporated into the scaffolds [39,46,47]. They significantly inhibited the growth of pathogens in planktonic culture and biofilm in a dose-dependent manner. For example, DOX released from CS enhanced calcium phosphate cement (CPCC) could reduce the colony-forming unit (CFU) of *Staphylococcus aureus* (*S. aureus*) and *P. gingivalis* by four orders of magnitude in biofilm [46].

Growth factor or stem cells improve osteoinduction and osteogenesis activity of tissue engineering scaffolds. These scaffolds had a porous structure, which is beneficial for growth factor loading, cell attachment, growth and proliferation [48]. Recombinant amelogenin (rhAm), an active ingredient to promote periodontal regeneration, was incorporated into a mesoporous hydroxyapatite/CS composite scaffold [39]. In the scaffold, sustained release of rhAm promoted osteogenic differentiation of hPDLCs. Animal experiments showed that the scaffold could promote the formation of cementoid tissue. Qiu et al. combined human periodontal ligament stem cell (hPDLSCs) beads with DOX to construct anti-infective bone regeneration scaffolds [46]. hPDLSCs could be released slowly from the beads and enhanced osteogenic differentiation. In addition, the released DOX not only enhanced calcium phosphate cement, but also inhibited bone resorption by inhibiting matrix metalloproteinases. Human periapical cyst mesenchymal stem cells (hPCy-MSCs), which were isolated from periapical cysts, were seeded on mineral-doped porous scaffolds [48]. The scaffolds enabled the reuse of biological waste and had the potential to regenerate bone and dental tissues. Apart from osteogenic ability, some elements were able to promote angiogenesis as well [47].

Tissue engineering scaffolds have antibacterial activity, improved osteoinduction and osteogenesis abilities, as well as excellent mechanical property in vitro. They are expected to control inflammation via combating infections and enhance bone regeneration. To confirm the results, further research is needed to investigate their long-term in vivo performances. Moreover, attention should be paid to the usage of some potentially toxic substances [47].

**Table 1:** The summary of periodontal biomaterials.

Biomaterials	Characteristics	Performances		Advantages	Limitations	Refs.
		In Vitro	In Vivo			
Bioactive agents	Biological or synthetic agents with both antibacterial and osteogenic effect	Antibacterial effect on *Porphyromonas gingivalis* (*P. gingivalis*) and *Streptococcus mutans* (*S. mutans*) Promotion of osteogenic differentiation of human periodontal ligament fibroblasts (hPDLFCs) and human periodontal ligament cells (hPDLCs)	Reduction in bone loss in animal model Probing depth (PD) reduction, clinical attachment level (CAL) gain and percentage of radiographic defect depth reduction (DDR%) in a clinical trial	Safety, abundant sources	Unknown active ingredient and mechanism	[8,11,14,15,49]
Guided tissue regeneration/guided bone regeneration (GTR/GBR) membranes	Composite membranes based on natural or synthetic polymers, combined with antibacterial and osteogenic components They act as cell barriers to prevent the invasion of epithelium and connective tissues, facilitating alveolar bone regeneration	Antibacterial effect on *P. gingivalis*, *S. mutans*, *Fusobacterium nucleatum* (*F. nucleatum*), *Staphylococcus aureus* (*S. aureus*), *Escherichia coli* (*E. coli*), *Streptococcus sanguis*, *Micrococcus luteus*, *Salmonella typhimurium*, *Peptostreptococcus anaerobius* (Pa), *Actinobacillus actinomycetes* (Aa) and *Enterococcus faecalis* (*E. faecalis*) Promotion of mineralization, proliferation, osteogenic differentiation of osteoblasts, fibroblasts, hPDLCs, bone marrow mesenchymal stem cells (BMSCs), Mg63 cells, MC3T3 cells, human periodontal ligament fibroblasts (HPDLFs), mesenchymal stem cells (MSCs), human periodontal ligament stem cells (hPDLSCs) and dental pulp stem cells (DPSCs) Induction of M2 phenotype polarization of macrophages	Reduction in bone loss, promotion of mucosal healing, new bone formation and fibril matrix deposition in animal model CAL gain, radiographic defect bone level (DBL) gain, reduction in PD and gingival recession (GR) in a clinical trial	Good biocompatibility, better mechanical property compared with traditional membrane, controllable biodegradability, drug encapsulation and release ability, manageability as well as accurate matching with the defects	Long degradation time, unknown long-term metabolic effect and toxicity of elements	[9,17,18,20,21,22,23,24,25,26,27,28,29,30,31,34,35,36,37,38,40,41,42]
Tissue engineering scaffolds	Scaffolds are composed of natural or synthetic polymers, antibacterial components, proteins or cells They are grafted to bone defect to maintain space, store growth factors, support cell attachment and proliferation	Antibacterial effect on *F. nucleatum*, *P. gingivalis*, *S. aureus* and *Aggregatibacter actinomycetemcomitans* (*A.actinomycetemcomitans*) Promotion of osteogenic differentiation of hPDLCs, hPDLSCs, promotion of osteogenic and odontogenic differentiation of human periapical cyst mesenchymal stem cells (hPCy-MSCs) and promotion of preosteogenic responses of MC3T3-E1 cells	Promotion of cementoid tissue formation in animal model	Good mechanical property, loading and sustained release of active ingredients, antibacterial property, improved osteoinduction and osteogenesis performances	Potential toxicity of certain ingredient, lack of research on osteogenic ability in vivo	[39,46,47,48]
Drug delivery systems	Natural or synthetic polymers are loaded with antibacterial and osteogenic components Along with systems’ degradation, active components experience burst release followed by sustained release	Antibacterial effect on *A. atinomycetemcomitans*, *Prevotella nigricans* (*P. nigrescens*), *E. coli*, *S. aureus*, *P. gingivalis*, *E. faecalis*, *S. mutans* and *Streptococcus sanguinis* (*S. sanguinis*) Promotion of proliferation, migration, attachment and osteogenic differentiation of human bone marrow-derived osteoblasts (HOB), BMSCs, osteoblasts and PDLSCs, promotion of mineralization	Reduction in bone loss, increase in new bone formation and improvement of gingival index in animal model	Stability, stimulus responsiveness, effective controlled release mode, long-lasting effects	Potential toxicity of certain ingredients	[11,13,50,51,52,53,54]

### 2.4. Drug Delivery Systems

Medication is an adjuvant to consolidate periodontal treatment and prevent the progression or recurrence of periodontal disease. Conventional periodontal medication has many limitations, including single effects, short-acting, drug resistance, side effects and inconvenient operation [55,56]. Furthermore, it does not show significant clinical and anti-microbial effects [57]. Currently, local drug delivery systems for the periodontal region are constructed by loading different substances with biocompatible carriers. These systems improve bioavailability of drugs, avoid side effects of systemic medication and achieving targeted, continuous and controllable release. Therefore, they are expected to be an ideal therapy [29,36]. 

The performance of drug delivery systems is related to type and proportion of the loaded components and surface of the carriers. In these systems, antibacterial components are not only antibiotics [51,54], but also metal nanoparticles such as silver [11,23,24,25,26,27,28,29,30,31,34,35,36,37,38,39,43,44,46,52,53,57,58] and polymer nanoparticles such as CS (nCS) [53]. Osteogenic components include drugs such as lovastatin and minocycline [51,54], metal nanoparticles such as silver and calcium [52,59], and polymer nanoparticles such as poly(lactic-co-glycolic acid) (nPLGA) and nCS [53], etc. Different ratios of active ingredients produce different effects. Xue et al. found that the performance of the mixture was the best when the nPLGA to nCS ratio was 3:7, and the content of silver nanoparticles (nAg) was 50 µg/mL [53]. In addition to the active components, surface modification of the carrier has direct contact with antibacterial properties. It kills bacteria by changing their membrane permeability without increasing drug resistance [59]. As the result, the systems have antibacterial activity against many kinds of bacteria and nearly achieve complete bone regeneration [13].

The mechanism of drug release from polymer includes diffusion and polymer degradation [54]. Thus, most systems present initial burst release and subsequent stable slow release for about 5–30 days. Initial burst release of antibiotics above minimal inhibitory concentration (MIC) is necessary since the control of infection and inflammation is more evident in the early stages of wound healing [29,50]. The subsequent stable release prevented the growth of residual bacteria [30]. In addition, infection must be controlled before bone regeneration. Therefore, Lee et al. designed a sequential release system which allowed the outside tetracycline release at the beginning, followed by the inside lovastatin [54]. This release strategy may increase the utilization of both antibacterial and osteogenesis ingredients. Furthermore, prolonged release time of lovastatin had a significant effect on enhancing periodontal bone regeneration.

**Figure 1:**
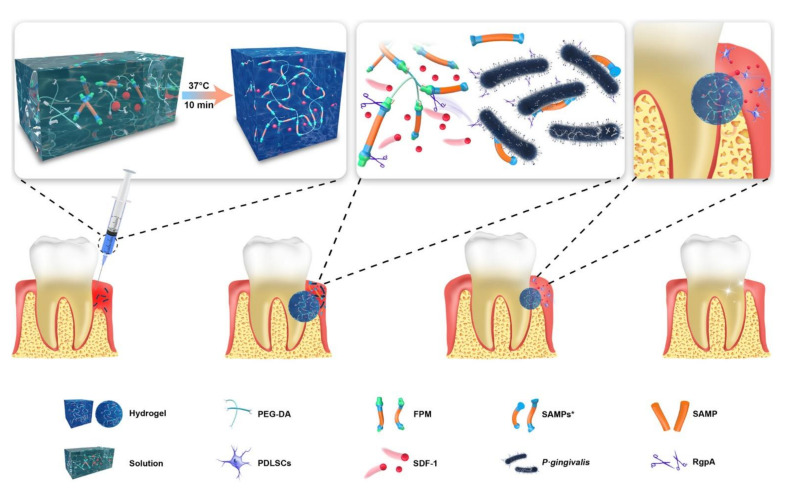
Schematic illustration of the composition and application of PEGPD@SDF-1 hydrogel. The hydrogel is cross-linked by FPM and PEG-DA and loaded with SDF-1. The transition from solution to hydrogel is completed at 37 °C in 10 min. FPM contains SAMP in the center and two anchor peptides with specific splicing sites in the lateral. The RgpA released by *P. gingivalis* initiates splicing of the specific sites in FPM. Then, SAMPs* and SDF-1 are released to resist *P. gingivalis* and promote osteogenesis, respectively. Reprinted with permission from reference [13]. Copyright 2021 American Chemical Society.

To improve drug targeting and achieve on-demand delivery in specific microenvironments, stimuli-responsive release has become a new trend. There are a lot of alkaline phosphatases (ALP) in the periodontal pocket of periodontitis patients, which can decompose polyphosphate esters (PPEs). Considering this, Li et al. added PPE and minocycline hydrochloride (MH) to CS to prepare an enzyme-responsive drug release film [51]. Within 12 days, the accumulative release of MH reached 80% under the action of ALP. In addition, MH provided effective antibacterial activity for the membrane. Compared with the control group, more human gingival fibroblasts (HGFs) and rat osteoblasts attached to the membrane, and the osteogenesis of rat osteoblasts was promoted. In vivo experiments confirmed that the membrane could improve gingival index and reduce alveolar bone loss. In addition, PEGPD@SDF-1 thermosensitive hydrogel loaded with antimicrobial peptide and stromal cell-derived factor-1 (SDF-1) was synthesized to control drug release according to the severity of infection (Figure 1) [13]. In the hydrogel, drugs were released in response to gingipain secreted by *P. gingivalis* and realized antibacterial properties as well as regeneration. With thermosensitive injectable characteristics, the hydrogel could accurately fit the defect with tiny or irregular shapes and avoid invasive operations. Continuous secretion of gingival crevicular fluid and oral movement make the periodontal environment unstable. Nevertheless, hydrogels with certain adhesion properties may remain stable and present constant function in the periodontal pocket. Furthermore, the porous structure of the hydrogels in accordance with bone compartment facilitated the penetration, growth and differentiation of stem cells.

Drug delivery systems have many advantages as well as limitations. Compared with conventional medication, they have an effective controlled release mode, long-lasting effects, good osteogenic potential and antibacterial activity [54]. In addition, more researchers have applied components with strong antibacterial effect, low bacterial resistance and weak cytotoxicity [13]. However, some ingredients are still toxic to cells at high concentrations [50,53,54]. Therefore, attention should be paid to adjusting their concentrations to achieve a balance between biological activity and biocompatibility. Furthermore, a drug release time suitable for both infection control and osteogenesis still needs to be determined.

## 3. Conclusions

Apart from excellent antibacterial and osteogenic effects, customization according to patients and lesions and the addition of growth factors and/or stem cells offer periodontal bifunctional biomaterials as a future potential for treatment of periodontitis. However, the time sequence of inflammation and bacteria in the development of periodontitis is still controversial. Recently, inflammatory continuum has been proposed as the driver. Therefore, a limitation of this review is the lack of exploration of anti-inflammatory biomaterials. In addition, since recent studies may emphasize a single property of certain bifunctional biomaterials, the materials in our review may be limited. Because of the risk of selection bias and the lack of evaluation of the validity of studies, readers should refer to this review with caution when selecting biomaterials.

## Data Availability

Not applicable.
